# Rosmarinic Acid as A Flavonoids Ameliorated Cytokines and Oxidative Stress Biomarkers in the Lung Lavage Fluid of Rats

**DOI:** 10.1002/fsn3.71442

**Published:** 2026-01-09

**Authors:** Vahideh Abbasnia, Mohsen Foadoddini, Mohammad Reza Khazdair

**Affiliations:** ^1^ Department of Biology Payame Noor University Tehran Iran; ^2^ Cardiovascular Diseases Research Center Birjand University of Medical Sciences Birjand Iran

**Keywords:** allergic asthma, bronchoalveolar lavage fluid, inflammation, oxidative stress, rosmarinic acid

## Abstract

Allergic asthma is a chronic respiratory condition characterized by pronounced inflammation, oxidative stress, and reversible airway obstruction. The objective of this study was to assess the effects of rosmarinic acid (RosA) on the mitigation of inflammation and oxidative stress in a rat model of asthma. Male Wistar rats were randomly assigned to six groups: (1) control group: administered normal saline via intraperitoneal (i.p.) and inhalation routes; (2) asthmatic group: exposed to ovalbumin (OVA) via i.p. and inhalation; (3) asthmatic rats treated with dexamethasone (1 mg/kg, orally); (4–6) three asthmatic groups receiving RosA at doses of 0.5, 1, and 2 mg/kg/day, orally. The levels of interleukin (IL‐4 and IL‐17A), interferon‐gamma (IFN‐γ), immunoglobulin E (IgE), nitrite (NO_2_), superoxide dismutase (SOD), catalase (CAT), malondialdehyde (MDA), and thiol (SH) were quantified in bronchoalveolar lavage fluid (BALF). Treatment with RosA significantly reduced the levels of IL‐4, IL‐17A, IgE, NO_2_, and MDA, while it elevated the levels of SH and IFN‐γ compared to the asthmatic group (*p* < 0.001). RosA dose‐dependently significantly elevated SOD and CAT activities compared to sensitized rats (*p* < 0.001). Furthermore, medium and higher doses of RosA significantly increased SOD and CAT activities compared to its lower dose (*p* < 0.001). The therapeutic effects of RosA on oxidative stress and inflammatory mediators in asthmatic rats were found to be comparable to those achieved with dexamethasone treatment. These results indicate the potential of RosA to elicit beneficial effects in the reduction of asthma symptoms.

## Graphical abstract

Levels of IL‐4, IFN‐γ and NO2 in broncho alveolar lavage fluid (BALF).

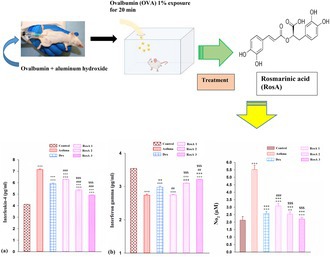

## 1 Introduction

The global incidence of asthma is increasingly concerning, currently impacting approximately 3% of the worldwide population and accounting for approximately 250,000 fatalities annually. Over 600 million individuals experience respiratory symptoms attributable to this condition. Asthma is a multifactorial disease that adversely influences both physical and psychological health, resulting in restricted physical capabilities and diminished overall quality of life (Gans and Gavrilova 2020).

Asthma pathogenesis is partial by environmental and genetic factors, with triggers that include allergens, infections, and irritants (Blumenthal 2012). There is considerable heterogeneity in the inflammation and remodeling of the respiratory pathway, processes that are mediated by the diffusion of cytokines and other inflammatory mediators (Aegerter and Lambrecht 2023). Notably, alterations in the ratios of interferon gamma (IFN‐γ) and interleukin 4 (IL‐4) signify a critical disturbance in the Th1/Th2 balance, which is essential in the progression of allergic syndromes such as asthma and allergic rhinitis (Zissler et al. 2016). Furthermore, oxidative stress is a substantial contributor to the pathophysiology of allergic reactions and the enrichment of inflammatory responses (Michaeloudes et al. 2022). This condition is particularly pronounced in individuals with asthma, correlating with exacerbations and chronic airway inflammation (Chung and Marwick 2010). Asthma disease often exhibits diminished antioxidant capacity, with minor levels of catalase (CAT), superoxide dismutase (SOD), and glutathione (Kirkham and Rahman 2006). Both endogenous and/or exogenous reactive oxygen species (ROS), as well as superoxide anion, the hydroxyl radical, nitric oxide, and nitrite, are critical in mediating airway inflammation (Sahiner et al. 2011).

Current asthma management primarily relies on anti‐inflammatory and bronchodilator medications specifically corticosteroids, β2‐agonists, and anticholinergics (Papi et al. 2020). However, conventional treatments present limitations; inhaled glucocorticoids may prove ineffective for certain patients and necessitate lifelong administration, while prolonged corticosteroid use can lead to harmful effects including diabetes, osteoporosis, and respiratory infections (Heffler et al. 2018). Recently, there has been a growing interest in herbal medicine as a treatment modality for various ailments (Cheng et al. 2012; Khazdair, Kianmehr, et al. 2020; Khazdair et al. 2021; Mortazavi Moghaddam et al. 2020). Clinical data indicate that natural products and herbal medicines are commonly better tolerated than their synthetic counterparts (Izzo et al. 2016; Khazdair, Ghorani, et al. 2020). Thus, identifying new therapeutic strategies that offer enhanced efficacy and/or reduced side effects remains a critical objective in asthma research.

Rosmarinic acid (RosA) is a polyphenolic compound found abundantly in several culinary herbs, including *Salvia rosmarinus* L. (rosemary), *Ocimum basilicum* L. (basil), and *Mentha arvense* L. (mint) (Luo et al. 2020). The pharmacological properties of RosA encompass anti‐inflammatory, antitumor, anti‐apoptotic, and antioxidant effects (Luo et al. 2020). In murine models of allergic asthma, RosA administration has been shown to significantly decrease the infiltration of inflammatory cells and the secretion of pro‐inflammatory cytokines. Additionally, RosA markedly diminished the production of ROS and substantially enhanced the activity of antioxidant enzymes (Liang et al. 2020). Research has also indicated that RosA significantly inhibits IgE‐mediated anaphylactic responses, as evidenced by a reduction in passive cutaneous anaphylaxis reactions and ear swelling in rat models (Jia et al. 2024).

Consequently, the present study aims to investigate the potential therapeutic effects of RosA on cytokine production and oxidative stress biomarkers in the lungs of sensitized rats.

## 2 Material and Methods

### 2.1 Animals

The male Wistar rats, weighing between 200 and 220 g, were utilized in this study under controlled conditions, maintaining a 12‐h light/dark cycle at a temperature of 22° ± 2°C. The rats had free access to food and water *ad libitum*. All experimental protocols adhered to the guidelines set forth by the National Institutes of Health for the care and use of laboratory animals and were approved by the Ethics Committee of Birjand University of Medical Sciences (IR.BUMS.REC.1402.489).

### 2.2 Experimental Groups

Animals were randomly allocated into the subsequent six groups (*n* = 8):

1 Control: animals were given normal saline via intraperitoneal (i.p.) and inhalation.
2 Sensitized: animals were given ovalbumin (OVA), i.p. and inhalation.
3 Dexamethasone (Dex): animals were given OVA (i.p. and inhalation) + Dex (1 mg/kg).
4‐6 RosA: animals were given OVA (i.p. and inhalation) + RosA at doses 0.5, 1, and 2 mg/kg, respectively in each group (Domitrović et al. 2014; Park et al. 2010; Sanbongi et al. 2004).

Drugs, including dexamethasone (Dex), RosA, and saline, were administered by gavage daily throughout the sensitization period for 21 days (Abbasnia, Eslimi Esfahani, et al. 2024).

### 2.3 Drugs

Aluminum hydroxide (Al (OH)3, CAS No.: 1330‐44‐5) and Ovalbumin (OVA, 98% pure; CAS No.: 9006‐59‐1) were obtained from Sigma Chemical (St. Louis, MO, USA). RosA (CAS No.: 20283‐92‐5) was purchased from Merck KGaA (Darmstadt, Germany), and dexamethasone (CAS No.: 50‐02‐2) was sourced from Abidi Pharmaceutical Co. (Tehran, Iran).

### 2.4 Sensitization Protocol

**FIGURE 1:**
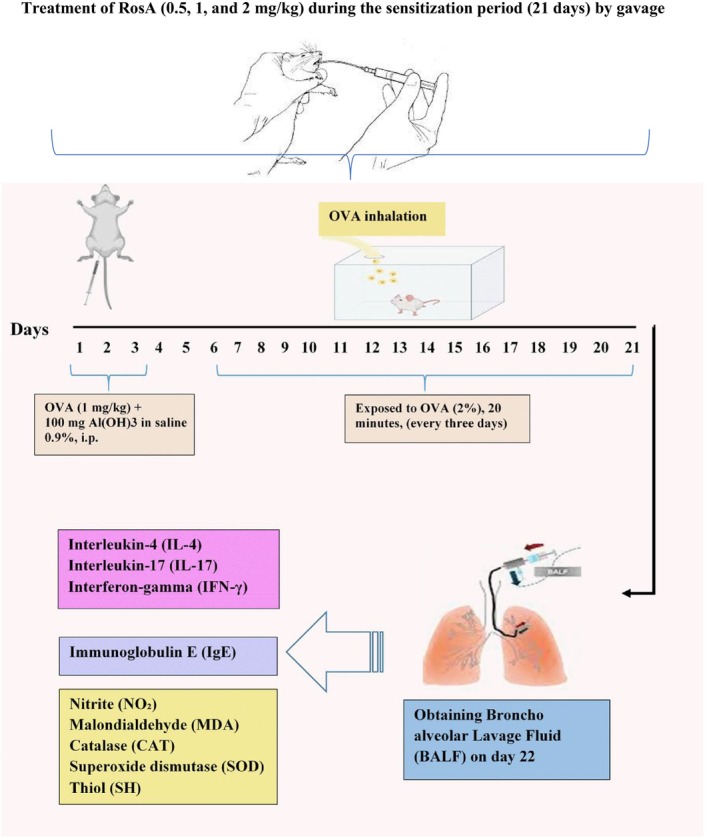
Ovalbumin (OVA) sensitization and protocol of the study.

Sensitization was induced by administering an OVA solution consisting of 1 mg/kg OVA combined with 100 mg aluminum hydroxide in 0.9% saline on Days 1, 2, and 3, intraperitoneally. From Days 6 to 21, rats were subsequently exposed to 2% OVA for 20 min every 3 days, as previously described (Abbasnia, Foadoddini et al. 2024). The timeline of the experiment is illustrated in Figure 1. Following the last challenge on Day 22, animals were anesthetized with ketamine hydrochloride (50 mg/kg, i.p.). The control group underwent the same procedures but received saline instead of OVA, as outlined in the previous study (Boskabady and Gholami Mahtaj 2015).

### 2.5 Preparation of Broncho Alveolar Lavage Fluid (BALF)

After sacrificing the animals, the lungs and section of the trachea were removed. The tracheae were cannulated, and lung lavage was performed by washing the lungs five times with 5 mL of saline solution. The collected BALF was then centrifuged at 2500 rpm at 4°C for 10 min. The supernatant was accumulated and stored at −70°C until further analysis.

### 2.6 Measurement of Cytokines, Immunoglobulin E and Oxidative Stress Factors in BALF

The levels of interleukin‐4 (IL‐4), (CAS No.: ab9811), interferon‐gamma (IFN‐γ), (CAS No.: ab239425), and immunoglobulin E (IgE) (CAS No.: ab99571) in the BALF were quantified using ELISA kits purchased from Abcam Inc. (Waltham, MA, USA). The level of IL‐17A was measured using IL‐17A ELISA kits (Catalog No.: KPG‐RIL17) from Karmania Pars Gene, (Rafsanjan, Iran).

Additionally, the levels of MDA were defined using an MDA Assay Kit (Catalog No.: KPG‐Nob), and NO_2_ levels were determined using a NO Assay Kit (Catalog No.: KPG‐NO). The activities of SOD and CAT were assessed using the SOD Activity Assay Kit (Catalog No.: KPG‐SOD) and Catalase Activity Assay Kit (Catalog No.: KPG‐CAT), respectively. All kits were obtained from Karmania Pars Gene (Rafsanjan, Iran). The total thiol group content was measured using a kit (Catalog No.: 101‐001‐2) from Kavosh Arya Azma, (Birjand, Iran). All measurements were performed according to the manufacturers' protocols utilizing a Bio‐Tek spectrophotometer (USA).

### 2.7 Statistical Analysis

The data are presented as mean ± standard error of the mean (SEM). Comparisons among the control, sensitized, and treatment groups were conducted using one‐way analysis of variance (ANOVA), followed by post hoc Tukey's test, with analyses performed using SPSS software version 23. A *p*‐value of less than 0.05 was considered statistically significant.

## 3 Results

### 3.1 Antibody and Cytokines Measurement

**FIGURE 2:**
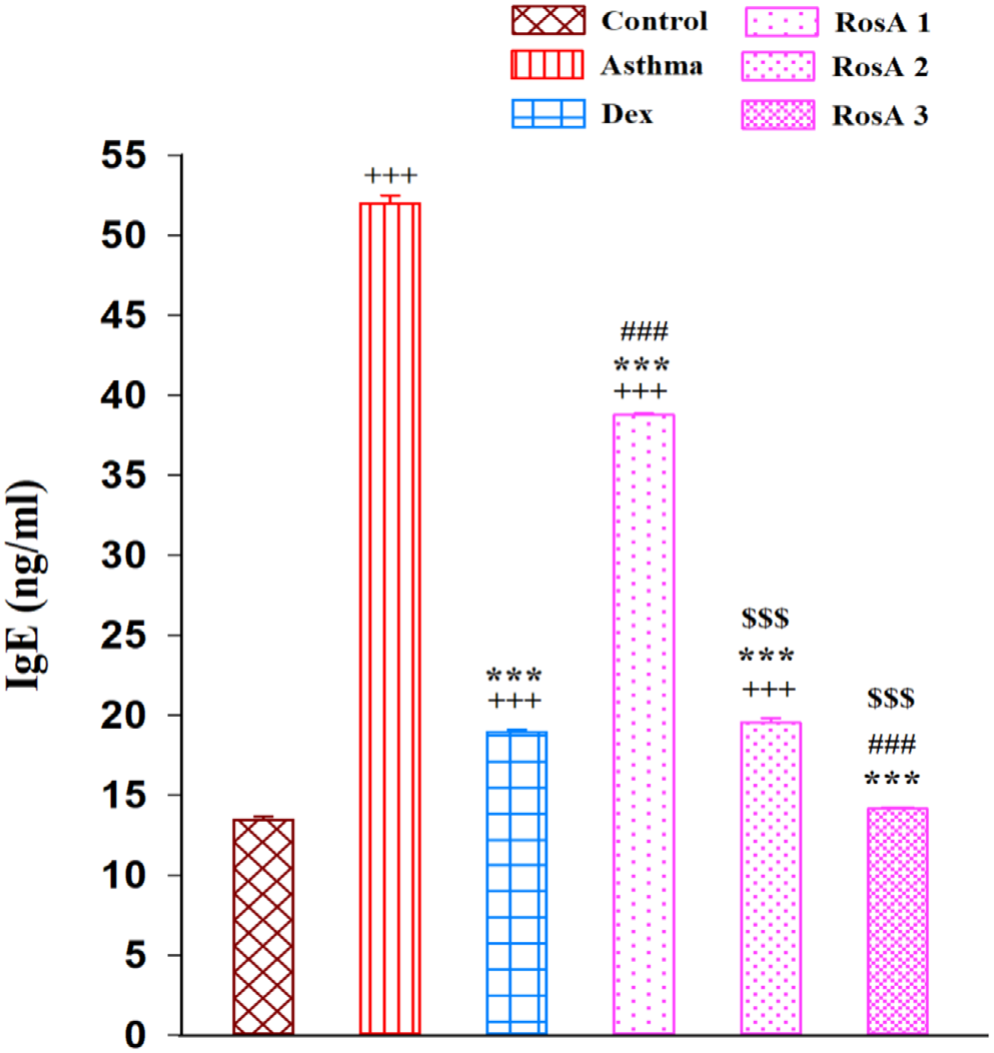
The mean ± SEM level of immunoglobulin E (IgE) in the BALF of experimental groups (*n* = 8). Comparison of the data between groups was done using one‐way analysis of variance (ANOVA) with Tukey–Kramer posttest. Dexamethasone (Dex); Rosmarinic acid (RosA). +++: *p* < 0.001, comparison between control and the other experimental groups. ***: *p* < 0.001, comparison between asthma and treatment groups. ###: *p* < 0.001, comparison between Dex and the other treatment groups. $$$: *p* < 0.001, comparison between RosA 0.5 vs. RosA 1 and 2 mg/kg.

The level of IgE in the BALF of the sensitized (asthma) group remarkably increased in comparison with the control group (*p* < 0.001). IgE concentration was meaningfully decreased in the treatment groups with Dex and three doses of RosA compared to the asthmatic group in a dose‐dependent manner (*p* < 0.001). The higher and medium doses of RosA significantly reduced the level of IgE compared to the low dose (0.5 mg/kg) treatment group (*p* < 0.001). Furthermore, the high dose of RosA (2 mg/kg) remarkably decreased the level of IgE compared to the Dex‐treated group (*p* < 0.001; Figure 2).

**FIGURE 3:**
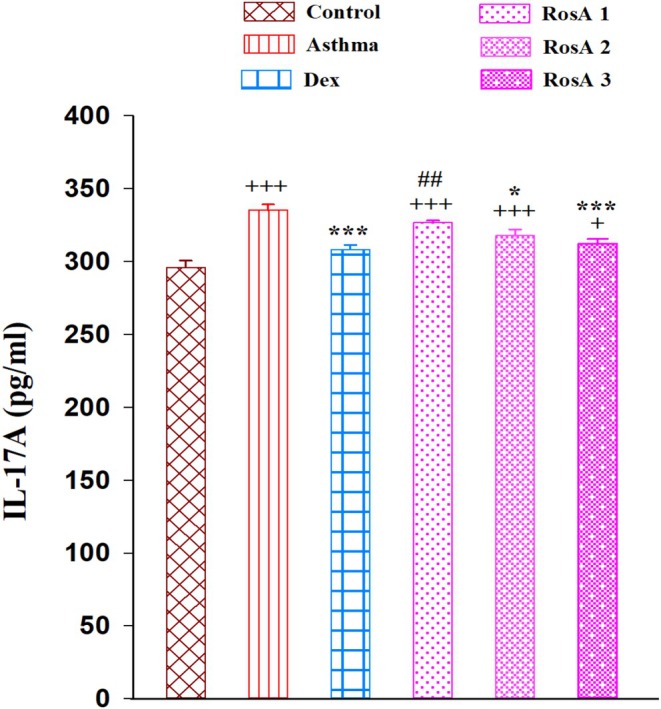
The mean ± SEM level of interleukin‐17 (IL‐17) in the BALF of experimental groups, (*n* = 8). Comparison of the data between groups was done using one way analysis of variance (ANOVA) with Tukey–Kramer posttest. Dexamethasone (Dex); Rosmarinic acid (RosA). +++: *p* < 0.001, comparison between control and the other experiment groups. *: *p* < 0.05 and ***: *p* < 0.001, comparison between asthma and treatment groups. ##: *p* < 0.01, comparison between Dex and the other treatment groups.

Concentration of IL‐17A in the BALF of the asthmatic rats was meaningfully increased in comparison with the control group (*p* < 0.001). Treatment with Dex, medium, and higher doses of RosA significantly reduced levels of IL‐17A compared to the asthmatic rats (*p* < 0.05 to *p* < 0.001; Figure 3). Although IL‐17A concentration in the low dose of RosA was lower than in the asthmatic group, there was no substantial difference.

**FIGURE 4:**
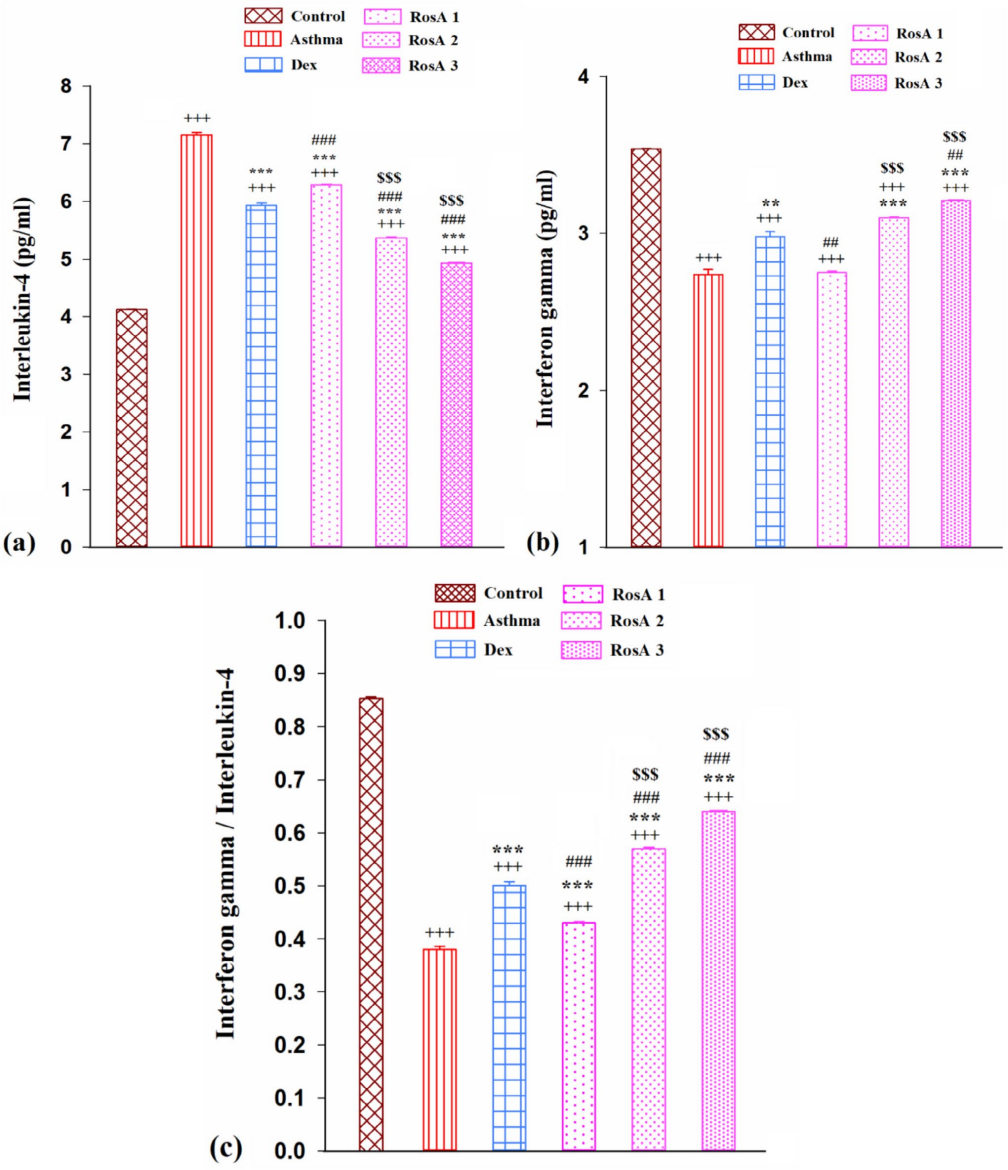
The mean ± SEM level of interleukin‐4 (a), interferon‐gamma (b) and IFN‐γ/IL4 ratio (c) in the BALF of experimental groups (*n* = 8). Comparison of the data between groups was done using one way analysis of variance (ANOVA) with Tukey–Kramer posttest. Dexamethasone (Dex); Rosmarinic acid (RosA). +++: *p* < 0.001, comparison between control and the other experiment groups. **: *p* < 0.01 and ***: *p* < 0.001, comparison between asthma and treatment groups. ##: *p* < 0.01 and ###: *p* < 0.001, comparison between Dex and the other treatment groups. $$$: *p* < 0.001, comparison between RosA 0.5 vs. RosA 1 and 2 mg/kg.

The measurement of IL‐4 level in the BALF showed an important rise in the asthmatic group in comparison with the control group (*p* < 0.001). However, treatment with Dex and three doses of RosA significantly decreased the level of IL‐4 compared to the asthmatic rats (*p* < 0.001, for all groups). Concentration of IL‐4 in the medium and high doses of RosA treatment groups was significantly lower than Dex and low dose treatment groups (*p* < 0.001, for both groups), (Figure 4a).

Concentration of IFN‐γ in the BALF (Figure 4b) and its ratio to IL‐4 (Figure 4c) significantly decreased in the asthmatic group in comparison with the control group (*p* < 0.001, for both cases). The level of IFN‐γ in the Dex, medium, and high doses of RosA treatment groups remarkably enhanced in comparison to the asthmatic group (*p* < 0.01 and *p* < 0.001) as well as significantly increased in medium and high doses of RosA compared to the low dose of RosA treatment rats (*p* < 0.001; Figure 4b).

The IFN‐γ/IL‐4 ratio was also significantly reduced in all sensitized (treated and untreated) groups (*p* < 0.001). However, treatment with Dex and all doses of RosA meaningfully increased the ratio of IFN‐γ/IL‐4 compared to the asthmatic group (*p* < 0.001). This ratio was significantly increased in the medium and high doses of RosA treatment groups compared to the Dex and low dose treatment groups (*p* < 0.001; Figure 4c).

### 3.2 Oxidative Stress Measurement

**FIGURE 5:**
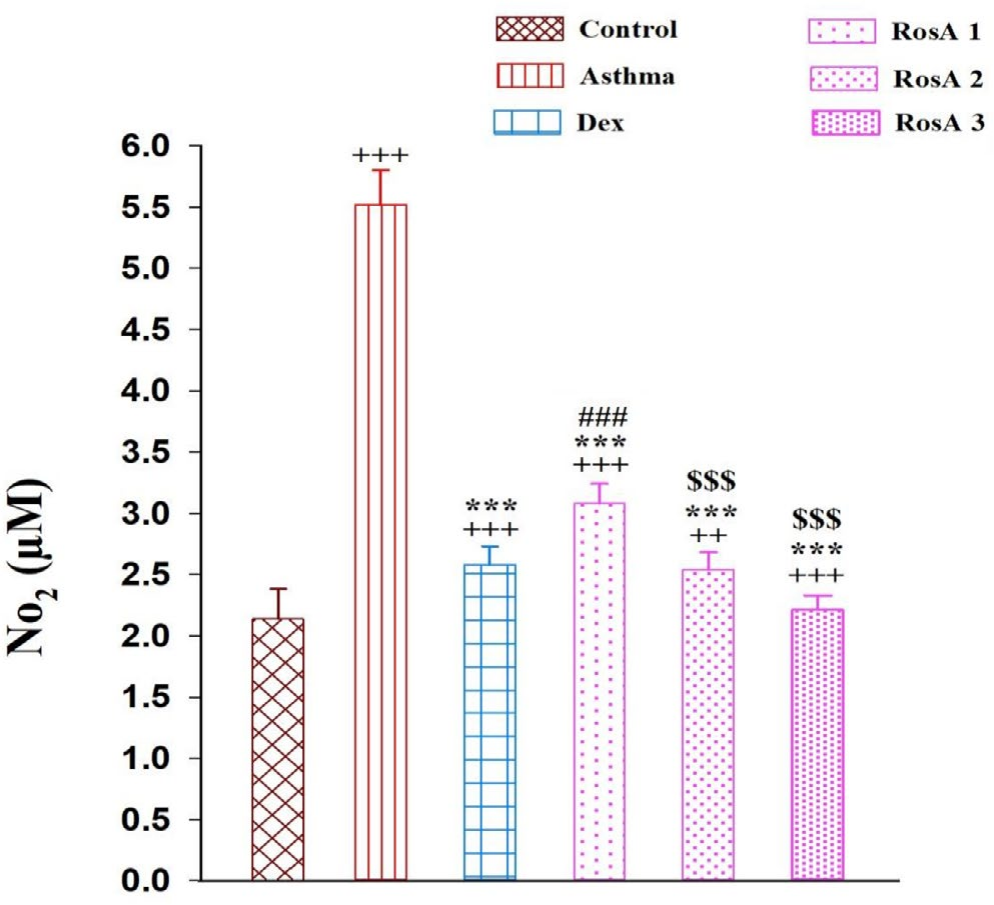
The mean ± SEM level of nitrite (NO_2_) in the BALF of experimental groups, (*n* = 8). Comparison of the data between groups was done using one‐way analysis of variance (ANOVA) with Tukey–Kramer posttest. Dexamethasone (Dex); Rosmarinic acid (RosA). ++: *p* < 0.01 and +++: *p* < 0.001, comparison between control and the other experimental groups. ***: *p* < 0.001, comparison between asthma and treatment groups. ###: *p* < 0.001, comparison between Dex and the other treatment groups. $$$: *p* < 0.001, comparison between RosA 0.5 vs. RosA 1 and 2 mg/kg.

**FIGURE 6:**
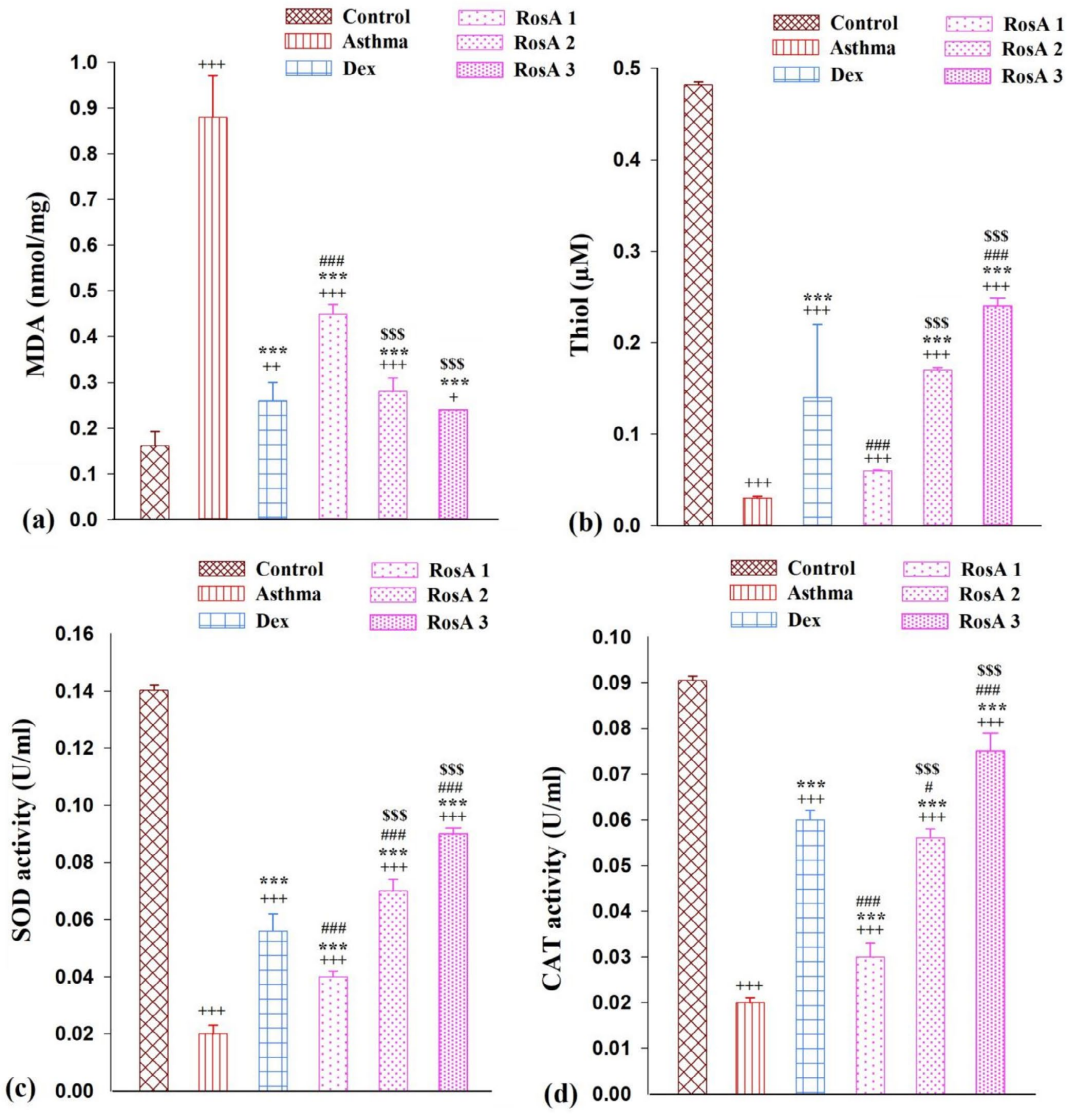
The mean ± SEM level of MDA (a), thiol (b), activities of SOD (c) and CAT (d) in the BALF of experimental groups, (*n* = 8). Comparison of the data between groups was done using one way analysis of variance (ANOVA) with Tukey–Kramer posttest. Dexamethasone (Dex); Rosmarinic acid (RosA). +: *P* < 0.05, ++: *p* < 0.01 and +++: *p* < 0.001, comparison between control and the other experiment groups. ***: *p* < 0.001, comparison between asthma and treatment groups. #: *p* < 0.05 and ###: *p* < 0.001, comparison between Dex and the other treatment groups. $$$: *p* < 0.001, comparison between RosA 0.5 vs. RosA 1 and 2 mg/kg.

The levels of NO_2_ and MDA meaningfully enhanced, while the levels of SH, SOD, and CAT activity were remarkably reduced in the asthmatic group in comparison with the control group (*p* < 0.001). Then, treatment with Dex and three doses of RosA significantly decreased levels of NO_2_ and MDA in comparison with the asthmatic group (*p* < 0.001; Figures 5 and 6a, respectively). The levels of the thiol group, CAT, and SOD activity were significantly higher in the Dex and all doses of RosA treatment groups in comparison with the control group (*p* < 0.001; Figure 6b–d, respectively). Interestingly, the levels of thiol in the high dose of RosA, and CAT and SOD activity in medium and higher doses of RosA were remarkably higher in comparison with Dex treatment animals (*p* < 0.05 to *p* < 0.001). Additionally, the medium and higher doses of RosA on oxidative stress biomarkers are significantly more effective than the low dose of RosA treatment group (*p* < 0.001; Figure 6).

## 4 Discussion

Activation of mast cells, eosinophils, and T‐helper 2 lymphocytes (Th2) produces inflammatory cytokines, including IL‐4 and IL‐13, that lead to respiratory inflammation in allergic asthma (Larché et al. 2003). Current therapeutic strategies for asthma primarily are based generally on the suppression of inflammation and alleviating the symptoms with inhalation corticosteroids and bronchodilators, respectively. However, these treatments do not consistently resolve airflow obstruction and inflammation in patients with asthma (Lemanske Jr and Busse 2010). The inflammatory cascade in allergic asthma is influenced by an imbalance between Th1 and Th2 immune responses. This imbalance exacerbates the inflammatory process by reducing the level of IFN‐γ and increasing the level of IL‐4 (Lambrecht et al. 2019). Moreover, IL‐17 is a potent mediator that collaborates with other cytokines, such as TNF‐α, to initiate and amplify pro‐inflammatory responses (Scichilone et al. 2013). In the previous study, treatment with RosA significantly reduced total white blood cell counts and the percentage of eosinophils in sensitized rats. Additionally, histological changes (muscle hypertrophy, inflammation, and mucus plaques) were significantly improved in the treated asthmatic groups (Abbasnia, Eslimi Esfahani, et al. 2024).

The outcomes of the current study showed that treatment of sensitized asthmatic rats with RosA decreased levels of antibodies and inflammatory mediators, notably IL‐4, IL‐17A, and IgE, in lung tissue. These findings suggest that RosA may play a beneficial role in modulating immune responses and ameliorating airway inflammation in allergic asthma.

The immunomodulatory effects of RosA on respiratory allergies induced by the *Blomia tropicalis* (Bt) mite in A/J mice showed remarkable reductions in total inflammatory cells and eosinophils, as well as decreased eosinophil peroxidase activity and IL‐4 levels in BALF (Costa et al. 2012).

Similarly, the preventive activities of RosA in female BALB/c mice induced asthma demonstrated that pretreatment with RosA (5–20 mg/kg) 1 h prior to the OVA challenge significantly inhibited increases in Th2 cytokines (IL‐4, IL‐5, and IL‐13) and markedly reduced IgE concentrations in the BALF (Liang et al. 2016). Additionally, administration of RosA significantly diminished the levels of phospholipase A2, IgE, IL‐4, and total protein in the BALF of OVA‐induced rats (Shakeri et al. 2019). These findings corroborate the results observed in the present study. It has been reported that treated sensitized mice challenged with OVA and H_2_O_2_ with RosA reduced the inflammatory cell numbers and production of IL‐4, IL‐5, and IL‐13, while augmenting the production of IFN‐γ (Liang et al. 2020).

Treatment of rat models of allergic rhinitis after particulate matter (PM2.5) exposure with RosA (20 mg/kg, i.p.) for 7 days remarkably reduced the levels of IL‐4 and IL‐13, while enhancing IFN‐γ levels in nasal lavage fluid. Moreover, treatment with RosA also significantly decreased the expression of GATA binding protein 3 mRNA, as a key transcription factor involved in immune and inflammatory responses, as well as the nuclear factor kappa B in induced rats (Zhou et al. 2022). The possible mechanism of RosA in improving allergic asthma by the gut‐lung axis has also been investigated. Short‐chain fatty acids upregulated duodenal monocarboxylate transporters, thus refining their systemic delivery to reduce Th2/innate lymphoid cells group 2 (ILC2s), mediated inflammatory responses and suppresses mucus production and the recruitment of eosinophils in the lungs. Notably, RosA increased SCFA to reduce Th2/ILC2‐mediated inflammatory and oxidative stress pathway in the lungs (Guo et al. 2024).

The effects of RosA on OVA challenge allergy in mice effectively improved the expression of anti‐inflammatory mediators, such as IL‐10 and forkhead box protein P3 (FOXP3). Meanwhile, it significantly inhibited the promotion of pro‐inflammatory mediators, including TNF‐α, IL‐4, IL‐6, mucosal mast cell protease‐1 (mMCP‐1), and iNOS in the liver of mice (Jia et al. 2023). In another similar study, administration of RosA in OVA‐induced intestinal allergy significantly reduced levels of anaphylactic mediators, such as OVA‐specific IgE, mMCP‐1, and histamine in female BALB/c mice. Additionally, RosA significantly inhibited Th2 cytokine expression while markedly increasing the levels of Th1 and regulatory T cell (Treg) cytokines (Yang et al. 2024). These findings from various animal studies support the current investigation regarding the therapeutic potential of RosA for improving lung inflammation. Numerous studies have attributed the anti‐asthmatic properties of natural products to their ability to decrease inflammatory mediators and improve oxidative stress in both in vitro and in vivo settings (Khazdair et al. 2019; Kianmehr and Khazdair 2020). It has been reported that administration of carvacrol (a monoterpenic phenol) in sulfur mustard‐exposed patients significantly increased anti‐inflammatory cytokines, reduced pro‐inflammatory cytokines, as well as improved pulmonary function test values in a randomized clinical trial (Khazdair and Boskabady 2019).

In the present study, the levels of sulfhydryl (SH) content, CAT and SOD activities as antioxidant factors were notably increased but MDA and NO_2_ levels significantly reduced in RosA treated groups compared to the untreated asthmatic group. Antioxidant effects of RosA in a mouse model of lungs damage induced by OVA and H_2_O_2_ significantly downregulated ROS production and significantly upregulated SOD, glutathione peroxidase (GPx), and CAT activities, accompanied by a reduction in the expression of NADPH oxidase (NOX)‐2 and NOX‐4 in lungs tissues (Liang et al. 2020). Furthermore, treatment with RosA at a dose of 5 mg/kg in rats exposed to electromagnetic fields significantly increased total antioxidant capacity levels. The levels of MDA and Sertoli cell apoptosis remarkably reduced in the RosA treatment group compared to the control (Hajhosseini et al. 2013). RosA also demonstrated a protective effect against acetaminophen‐induced hepatotoxicity in rats, significantly decreasing aspartate aminotransferase and alanine aminotransferase levels while increasing SOD activity. Notably, RosA treatment also led to a decrease in TNF‐α expression and its regulator, receptor for activated C‐kinase 1, which is closely associated with oxidative stress (Yu et al. 2021). However, the reported anti‐asthmatic efficacy of RosA has not been uniformly consistent across all experimental settings. Available evidence indicates that the magnitude of RosA's immunomodulatory and antioxidant effects can be influenced by dose, route of administration, treatment duration, and the specific animal model and sensitization/challenge protocol employed (Liang et al. 2016; Luo et al. 2020). In line with this, several investigations have highlighted dose‐dependent responses, whereby lower doses may exert only modest effects on airway inflammation or selectively alter particular mediators without fully normalizing the broader inflammatory/oxidative profile (Liang et al. 2016; Shakeri et al. 2019). Moreover, differences in outcome measures (e.g., BALF cytokines vs. histopathology or airway hyperresponsiveness endpoints) may further contribute to variability among studies (Luo et al. 2020). Therefore, while the preponderance of evidence supports a protective role for RosA in allergic airway inflammation (Costa et al. 2012; Liang et al. 2020), these methodological sources of heterogeneity should be considered when interpreting its therapeutic potential. Notably, to date, clear detrimental or pro‐asthmatic effects of RosA have not been reported in the available preclinical literature (Luo et al. 2020).

The strength of the present study is a comprehensive BALF‐based evaluation of RosA in an established OVA‐induced rat asthma model, including a dose–response assessment and a direct comparison with dexamethasone under identical experimental conditions. This study is among the first to (i) simultaneously quantify Th_2_ and Th_1_ associated cytokines (IL‐4, IL‐17A, IFN‐γ) and IgE in BALF after RosA treatment, (ii) measure a full panel of oxidative stress markers (NO_2_^−^, MDA, SH, SOD, CAT) in BALF in this context, and (iii) compare RosA directly with a standard corticosteroid. These elements together provide new insight into the dual anti‐inflammatory and antioxidant actions of RosA and its potential translational relevance as a natural therapeutic candidate for allergic asthma.

Although the current study is limited by the absence of investigation into OVA‐specific IgE levels and the lack of direct mechanistic analyses, including flow cytometry and Western blotting to evaluate pathway‐specific proteins, it is crucial to address these limitations in future research to attain a more thorough understanding of the findings.

## 5 Conclusion

In conclusion, our findings indicate that RosA exerts a protective effect against OVA‐induced lung inflammation, likely through modulation of IgE levels, reduction of Th2 cytokine expression, and enhancement of Th1 cytokine production. Specifically, RosA appears to suppress oxidative stress responses, as evidenced by the reduction in MDA levels and enhancement of thiol content, CAT, and SOD activity. These results highlight the necessity for further basic and clinical studies to elucidate the protective effects of RosA for alleviating asthma symptoms.

## Author Contributions

**Vahideh Abbasnia:** concepulization, data curation, formal analysis, writing‐original draft. **Mohsen Foadoddini:** conceptulization, methodology, data curation, review and editing. **Mohammad Reza Khazdair:** conceptualization, funding acquisition, writing – original draft, writing – review and editing, formal analysis, supervision. All the authors approved the final version of the manuscript.

## Conflicts of Interest

The authors declare no conflicts of interest.

## Data Availability

The datasets used in the current study are available from the corresponding author on reasonable request.
